# Temporal dynamics of stream fish assemblages and the role of spatial scale in quantifying change

**DOI:** 10.1002/ece3.5954

**Published:** 2020-01-02

**Authors:** Zachery D. Zbinden

**Affiliations:** ^1^ Department of Biological Sciences University of Arkansas Fayetteville AR USA

**Keywords:** biodiversity monitoring, community stability, domains of scale, headwater streams, persistence, spatial clustering, spatial grain, temporal beta diversity

## Abstract

1. Spatial grain of studies of communities is often based on arbitrary convention. Few studies have examined how spatial scaling of grain size affects estimates of compositional change over time, despite its broad implications.
2. Fish assemblage structure was compared between 1974 and 2014 at 33 sampling locations in the Muddy Boggy River drainage, USA. The two main objectives for this comparison were to quantify change in assemblage structure and to test for a relationship between compositional change and spatial scale. Spatial scale was manipulated by pooling assemblage data into a continuous range of groups, which increased in size from *K* = 33 pairs (i.e., local scale) to *K* = 1 pair (i.e., global scale), via clustering algorithm based on pair‐wise fluvial distance.
3. Local assemblages (stream reaches) varied in the degree of assemblage change over time (range = 0.10–0.99 dissimilarity; mean = 0.66). The global assemblage (drainage), however, remained relatively similar. A discontinuity in the relationship between compositional change and spatial scale occurred at *K* = 15 (mean dissimilarity = 0.56; *p* = .062), and this grouping is roughly the size of the headwater/tributary drainages (i.e., stream order ≤ 3) in the study system.
4. Spatial scale can impact estimates of biodiversity change over time. These results suggest assemblages are more dynamic at individual stream reaches than at the scale of the entire drainage. The decline in assemblage change at the spatial scale of *K* = 15 deserves further attention given the marginal significance, despite a small sample size (*n* = 15). This pattern could suggest regional and meta‐community processes become more important in shaping assemblage dynamics at the scale of headwater drainages, whereas the factors responsible for driving individual stream reach dynamics (e.g., stochasticity) become less important. Defining assemblages at a larger scale will result in different estimates of species persistence. Biodiversity monitoring efforts must take the effect of spatial scaling into consideration.

## Graphical abstract

Estimates of stream fish assemblage change between two sampling periods 40 years apart were influenced by the spatial scale used to define assemblage size.

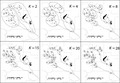

## 1 INTRODUCTION

Ecologists need nonarbitrary guidelines for defining the spatial scale of communities of interest, which is typically based more on convention than science (Frost, DeAngelis, Bartell, Hall, & Hurlbert, 1988; Jackson & Fahrig, 2015). One approach, for example, is to determine the minimum area in which a community is stable and/or persistent (Connell & Sousa, 1983). Another approach is to test for “domains of scale” (Wiens, 1989) or nonlinear relationships between some quantity of interest (e.g., species richness) and a scaling parameter (e.g., spatial grain), which would suggest a hierarchy within which generalizations could be made regarding causal factors acting at different domains (O'Neill, Deangelis, Waide, & Allen, 1986). Regardless of the approach, more thought must be given to objectively determining and justifying the scale at which data are collected—our inferences depend on it (McGill, 2010).

Spatial hierarchies or domains can be understood by investigating the relationship between rates of change in some parameter and scale. Discontinuities in the relationship between the parameter and scale, and/or peaks of unusually high variance, indicate where domains arise on the scaling axis (Greig‐Smith, 1979). The existence of separate domains is suggestive of a “boundary” at which the forces shaping the variation of the parameter of interest begin to shift from one set of driving forces to another. Despite decades of interest in hierarchy theory/domains of scale, few ecological studies have explicitly taken it into consideration (but see Estes et al., 2018; Viana & Chase, 2018; Wheatley, 2010).

Fields outside ecology were the first to recognize the importance of grain size on variation of measurements (Robinson, 1950) and that variation was usually highest when grain size was smallest (Meentemeyer, 1989). Pioneering simulation studies demonstrated a positive relationship between spatial heterogeneity and scale with population persistence (Paine & Levin, 1981; Reddingius & den Boer, 1970). These studies, in part, inspired subsequent focus on “landscape ecology” (Galzin, 1987; Turner, O'Neill, Gardner, & Milne, 1989) and meta‐populations (Hanski, 1994; Taylor, 1988)—eventually leading to the meta‐community concept (Leibold et al., 2004). The main conclusion of this body of work was that instability at local levels resulting in extirpations need not translate to instability at broader scales when sampling units are connected by dispersal. Furthermore, fine‐scale instability may even be requisite for stability at broader scales (DeAngelis & Waterhouse, 1987).

A rich literature exists on spatial dynamics of aquatic communities relating to longitudinal gradients (Perkin, Murphy, Murray, Gibbs, & Gebhard, 2019; Schlosser, 1987; Vannote, Minshall, Cummins, Sedell, & Cushing, 1980), as well as the effects of scale on community composition at one point in time (Geheber & Geheber, 2016; Paavola et al., 2006; Viana et al., 2016). However, relatively few studies have evaluated the influence of spatial scale variation on temporal dynamics of communities (Fuhlendorf & Smeins, 1996; Sirami, Brotons, & Martin, 2008), to say nothing of aquatic communities. Most of those examining fish assemblages specifically did not deal with spatial scale per se, but rather longitudinal gradients (i.e., stream hierarchy; Schlosser, 1982) differences among habitats (Bart, 1989) or among areas within a system (Geheber & Piller, 2012). The studies that have explicitly considered spatial scale typically compare two scales: “local” and “global” (Collins, 2000). Generally, greater persistence of communities is observed at the broadest, compared with the finest, spatial scale (Hitt & Roberts, 2012; Matthews & Marsh‐Matthews, 2016; Ross, Matthews, & Echelle, 1985). In addition, several studies have addressed differences in biotic homogenization at local compared with global scales (Baiser, Olden, Record, Lockwood, & McKinney, 2012; Marchetti, Lockwood, & Light, 2006; Olden, Kennard, & Pusey, 2008; Villeger, Blanchet, Beauchard, Oberdorff, & Brosse, 2011).

The purpose of the analyses presented here was to determine whether and where a transition between assemblage change driven by local factors and change driven by more regional processes occurs. Here, domains of scale hypothesis (sensu Wiens, 1989) are tested regarding temporal assemblage dynamics for freshwater stream fish. If domains of scale do exist, then the relationship between assemblage change and size of the spatial scale used to assess that change should be nonlinear (i.e., “breaks” in the continuous relationship will exist that represent the border of domains). If domains do exist, their spatial scale will serve to objectively define where a transition between change driven by local factors and change driven by more regional processes occurs. In addition, the spatial extent that maximizes persistence (i.e., minimizes compositional change) may serve as a better spatial extent for what is often arbitrarily deemed an assemblage. Monitoring biodiversity at such a scale would minimize the amount of change due simply to dispersal, random factors, and other natural processes that predominately affect smaller areas.

## 2 MATERIALS AND METHODS

### 2.1 Study site

The Muddy Boggy River in the Cross Timbers Ecoregion of southeastern Oklahoma, U.S.A., is a major tributary to the Red River draining 6,291 km^2^ from an area 113 km (north–south) by 48 km (east–west) (Pigg, 1977). There is little urban development and only modest industrial development and natural resource extraction. The three largest cities in the area had the following populations as of the 2010 U.S. census: Ada (16,810), Atoka (3,107), and Coalgate (1,967).

The river flows southeastward and is formed by the confluence of Clear Boggy Creek (west—HUC 11140104) and Muddy Boggy Creek (east—HUC 11140103). Geologically, the upper reaches are in the northern Arkoma basin between the Arbuckle mountains (west) and the Ouachita mountains (east). This upper section is more topographically variable, and streams are higher gradient than those in the Dissected Coastal Plains near the confluence with the Red River (Pigg, 1977). The higher gradient streams are generally clear, swift‐flowing, and contain riffle‐run‐pool structure with gravel substrate (Pigg, 1977; personal observation). Lower gradient streams to the south are more turbid and sluggish, and often have more homogenous, mud‐bottom‐channel habitat littered with coarse woody debris (Pigg, 1977; personal observation).

### 2.2 Data collection

Beginning in 1974, Jimmie Pigg made 277 fish collections in the drainage, and of these, 174 were made with seine nets and in streams, while the rest used gill nets or electroshocking equipment and/or were made in ponds or ditches (Pigg, 1977). In 2014, I made 65 fish collections in streams of the drainage (Zbinden & Matthews, 2017) during the same months (May–September) using the same gear (4.57 m × 1.22 m × 4.88 mm mesh seine) and methods (sampling all microhabitats with 100 m stream reach) as Pigg. Efforts were made to revisit the exact stream reaches sampled four decades earlier, but changes in land use and access were problematic. All sites (174 + 65) were mapped using latitude and longitude, and each location sampled in 2014 was paired with the nearest site from 1974 (by fluvial distance). Pairs were retained for analysis if the straight‐line distance between was less than eight kilometers and the stream order was the same for both sites. Similar approaches of comparing assemblages through time have been used previously (Matthews & Marsh‐Matthews, 2015). Eight of the sites sampled in 2014 were sampled again in 2015 during the same months to provide context for assemblage fluctuation over one year.

### 2.3 Summarizing diversity and quantifying change

All statistical analyses were performed with R version 3.4.1 (R Development Core Team, 2016). Prior to analyses, *Campostoma anomalum* and *Campostoma spadiceum* collected in 2014 were collapsed into the group *Campostoma* spp. because recognition of *C. spadiceum* did not occur until 2010 (Cashner, Matthews, Marsh‐Matthews, Unmack, & Cashner, 2010) and thus were not differentiated by J. Pigg. In the same manner, *Fundulus notatus* and *Fundulus olivaceus* were collapsed into *Fundulus* spp. due to identification issues in this region (J.F. Schaefer and W.J. Matthews, personal communication). Finally, individuals identified as *Notropis rubellus* in 1974 were reassigned to *Notropis suttkusi,* following Humphries and Cashner (1994).

Fish species abundance data from 1974, 2014, and 2015 were compiled into a single matrix, and dissimilarity among all pair‐wise combinations was quantified using Morisita–Horn index (Horn, 1966; Jost, Chao, & Chazdon, 2011; Matthews & Marsh‐Matthews, 2017; Morisita, 1959) with the R package “vegan” (Oksanen, 2015). In addition, the abundance matrix was transformed into a binary matrix of presence/absence data, and this was used to calculate Jaccard's Index. This was done to determine whether the data resolution would affect the pattern observed (abundance vs. presence/absence). Only distances between target pairs (i.e., the matching local sites from ′74 and ′14; ′14 and ′15) were extracted from the dissimilarity matrix (33 pairs plus 8 pairs).

Nonmetric multidimensional scaling (NMDS, Kruskal, 1964) was used to visualize the dissimilarity matrix in multivariate space. This visualization allows inspecting differences among local assemblage pairs and the differences among global “clouds” of assemblages from 1974 and 2014. In addition, NMDS containing two sets of samples from different time periods allows for visualization of parallel trajectories among sites within the drainage. Stress less than 0.20 was considered the threshold for accurate representation of the data (Kruskal, 1964).

To test for the effect of geographic distance between assemblages and time between collections, dissimilarity values were pooled into three groups: 1974–2014 pair‐wise comparisons from the precisely same location (*n* = 12); 1974–2014 pair‐wise comparisons between approximately matching locations (*n* = 21); and pair‐wise comparisons between 2014 and 2015 (*n* = 8, all exact matches). Linear regression was used to test the relationship between Morisita–Horn dissimilarity and geographic distance between appropriate site pairs (both straight‐line and fluvial distance).

Diversity of the global assemblage (33 sites pooled) was compared between 1974 and 2014 using a variety of measures including species richness (MacArthur, 1965) and Simpson's reciprocal diversity (Simpson, 1949) using the R package “diverse” (Guevara, Hartmann, & Mendoza, 2016). Alpha, beta, and gamma diversities were obtained via bootstrapping with 10,000 iterations with R package “vegetarian” (Charney & Record, 2015; Jost, 2007).

### 2.4 Analyzing spatial effects on assemblage change

At the finest spatial level, or the “local” level, the data set contains 33 pairs of sampling localities where fish were collected in 1974 and 2014. At the broadest spatial level, or the “global” level, the data contain 1 pair of pooled localities sampled in 1974 and 2014. The R package “ClustGeo” (Chavent, Kuentz‐Simonet, Labenne, & Saracco, 2018) was implemented to create a hierarchy based on spatial location to create the intermediate groupings between 33 pairs and 1 pair. A dendrogram of all sampling localities was created using a Ward‐like clustering algorithm. The algorithm requires two distance matrices as input, in this case: Morisita–Horn dissimilarity among sites and fluvial distance (river‐km) between sites. Alpha weight (1.0) was used as the mixing parameter so that the spatial matrix alone would be used to cluster the sites. Thus, all 33 sites were clustered based on spatial proximity to one another. A separate community matrix was created for each clustered group containing the pooled data of the sites within each group for 1974 and 2014. For example, for *K* = 2 there were four rows of species abundance data: 2 sites × 2 sampling periods. Just as described above, Morisita–Horn dissimilarity index matrices were calculated for each of the 33 community matrices. The compositional distances between 1974 and 2014 for each group within a clustered set were then extracted. So, for each group K there would be K number of distances. The distributions of the distances for each spatial cluster were visualized to inspect the relationship between the number site pools (i.e., spatial scale) and the compositional change over 40 years (MH‐index).

The visual inspection of the relationship was used to determine where potential “breaks” in the relationship may occur. I tested the hypothesis that the breaks did not differ from the local spatial scale (*K* = 33) using a bootstrapping procedure. First a distribution of means was created by sampling the MH‐dissimilarity values for the *K* = 33 spatial group. The number of samples taken for each iteration was set equal to the number of samples from the *K* group being tested (e.g., if *K* = 20 was tested, 20 samples from *K* = 33 would be selected out of the 33 possible). Samples were made with replacement, and the procedure was repeated 100,000 times to generate the test distribution. To test the null hypothesis that the K group being tested was not different from the *K* = 33 spatial group, the probability of sampling a group with ≤the mean of the *K* group—given the bootstrapped distribution—was calculated (i.e., *p*‐value). The type 1 error rate was set to *α* = 0.05.

## 3 RESULTS

**Figure 1:**
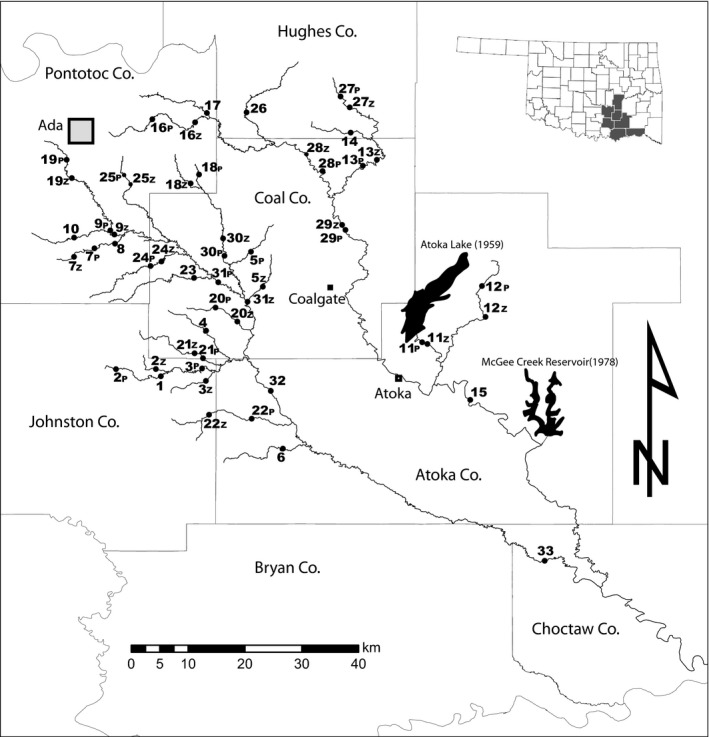
Map of sampling locations. If the locations were not identical reaches, each is denoted by a point and number followed by either a “P” for “Pigg 1974” or a “Z” for “Zbinden 2014”

Fish assemblages from 33 locations (Figure 1) sampled in 1974 and 2014 were compared. Local assemblages showed considerable variation in the degree of compositional change over time, but overall composition at the global level was little changed. There were 37 species common to both 1974 and 2014; in addition to these, 9 species were collected only in 1974, and 7 species were collected only in 2014 (See Table S1 for species list). All species not collected during both sampling periods were rare: occurring at only one site (*n* = 14) or at an abundance of one individual per site (*n* = 2), and therefore, it is likely they evaded capture during a sampling period rather than being either extirpated or colonizers. None of the global diversity indices differed between sampling periods: Simpson's Reciprocal Diversity (1974 = 4.23 ± 0.80 *SE* and 2014 = 3.95 ± 0.32), Alpha diversity (1974 = 9.48 ± 0.15 and 2014 = 10.79 ± 0.14), Beta diversity (1974 = 4.85 ± 0.20 and 2014 = 4.17 ± 0.11), and Gamma diversity (1974 = 46.0 ± 1.60 and 2014 = 45.0 ± 0.86).

**Figure 2:**
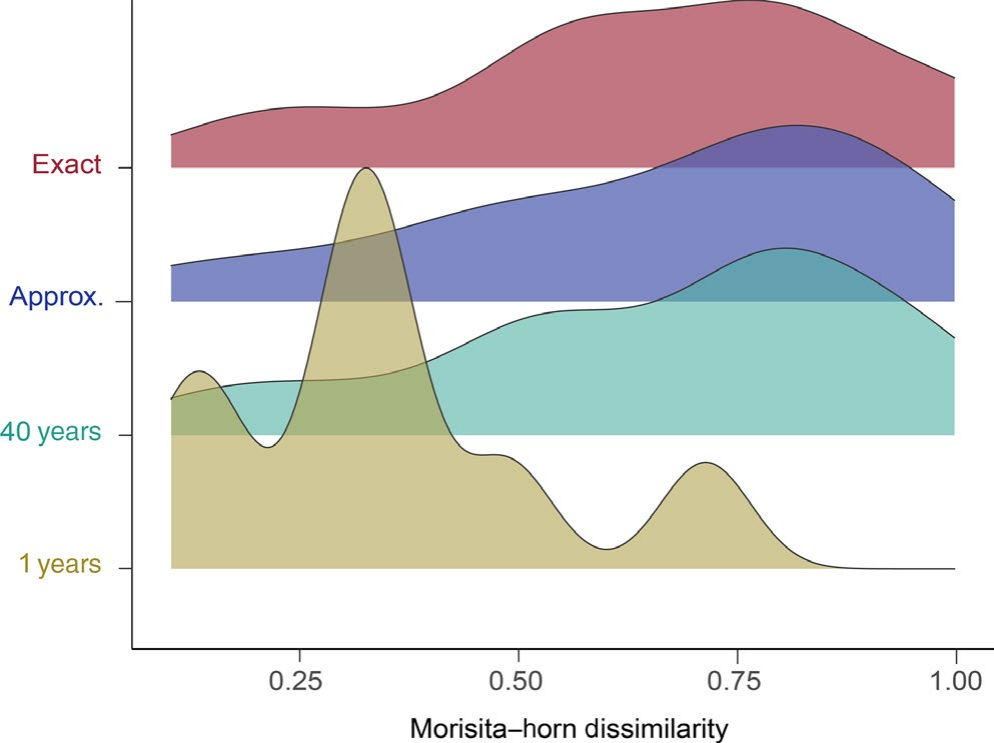
The distribution of compositional change among groupings of interest including (i) 40‐year compositional change in exact geographic matches, (ii) 40‐year compositional change in approximate geographic matches, (iii) all 40‐year comparisons together, and (iv) 1‐year compositional change between 2014 and 2015

Choice of dissimilarity index did not affect overall patterns or estimates of assemblage change. MH dissimilarity and Jaccard's Index were significantly correlated (*R^2^* = .167, slope = 0.670, *F*‐stat = 7.81, *n* = 41, *p* = .008) and did not statistically differ (*t*‐stat=−0.540, *n* = 41, *p* = .592). A summary is provided in Table 1, and only MH dissimilarity is presented hereafter. Local assemblages often changed drastically between 1974 and 2014 (Figure 2). Local assemblages ranged from 0.10 to 0.99 dissimilarity, and mean dissimilarity was 0.66 for the 33 pairs. Assemblages changed more between 1974 and 2014 than they did between 2014 and 2015 (mean = 0.66 and 0.35, respectively; *t*‐stat = 3.32, *n* = 8, *p* = .002; Figure 2). Assemblage dissimilarity did not differ between groups of exact location/same stream reach (mean = 0.66) and approximate location matches (mean = 0.66; *t*‐stat = −0.038, *n* = 12, *p* = .485; Figure 2), nor was dissimilarity for matching sites correlated with straight line (*R* ^2^ = .030, slope = −1.01, *F*‐stat = 0.943, *n* = 33, *p* = .339), or fluvial distance between sites (*R* ^2^ = .028, slope=−1.61, *F*‐stat = 0.885, *n* = 33, *p* = .354; Figure 2). This suggested the scheme used to select sites for comparison had not influenced the analyses.

**Table 1:** Summary of Jaccard and Morisita–Horn dissimilarity indexes for the 33 site comparisons between 1974 and 2014

	Jaccard	Morisita–Horn
Mean	0.665	0.660
*SD*	0.144	0.249
*SE*	0.025	0.043
Minimum	0.313	0.103
Median	0.667	0.741
Maximum	0.952	0.998
Fluvial Dist. *R* ^2^	.014	.028
Fluvial Dist. *p*	.509	.354

Note

Summary statistics of the distribution and results of linear regression between each index and fluvial distance among sites are presented.

**Figure 3:**
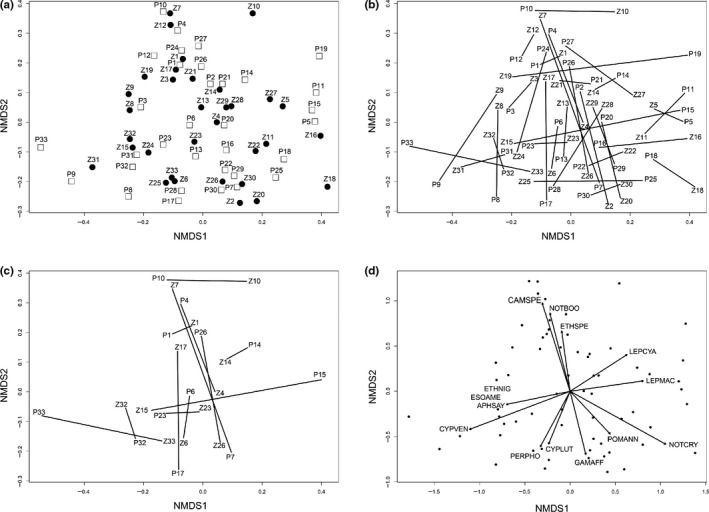
(a) NMDS of 33 assemblages from 1974 (white squares labeled “P”) and 33 assemblages from 2014 (black circles labeled “Z”). (b) The same NMDS from A, but with vectors illustrating assemblage change among sampling periods; (c) NMDS with only the 12 exact geographic matching sites; (d) ordination of NMDS with species significantly related to axes shown as vectors. Species identities are coded as the first three letters of the genus and species name. See Table S1 for species and species codes

Figure 3 (Panel A) illustrates how “clouds” of points from 1974 and 2014 were similar in location and spread. Panels B and C of Figure 3 demonstrate two points: First, assemblages varied in how much change occurred through time no matter if the sites were approximate matches (Panel B) or the same reach (Panel C), and second, there is no pattern of parallel trajectories that would suggest common shifts in composition across sites, or a trend toward global homogenization.

**Figure 4:**
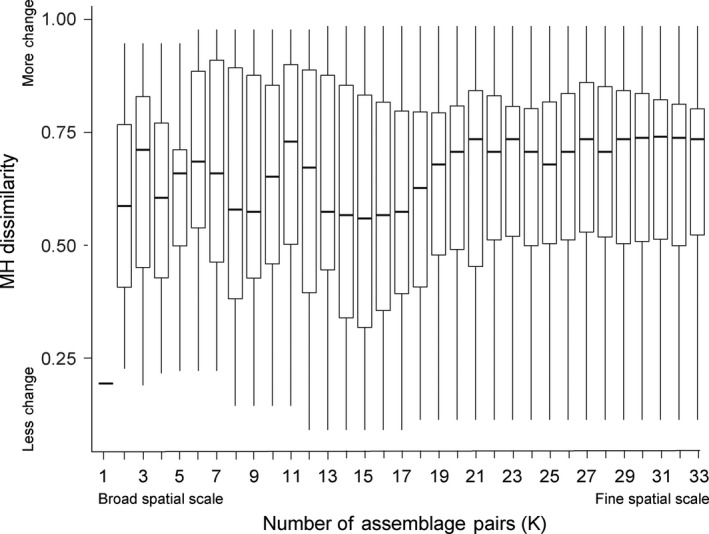
A boxplot showing the relationship between the number of the spatial clusters (*K*) and assemblage compositional change between 1974 and 2014 (Morisita–Horn dissimilarity) for the sites within the clusters

**Figure 5:**
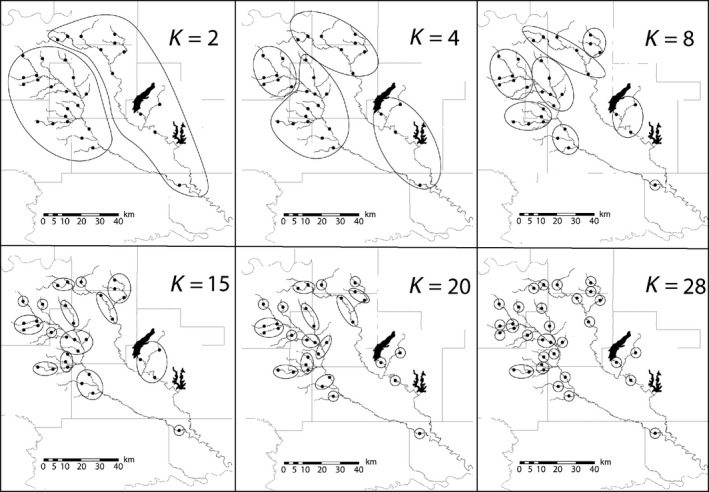
These six maps of the drainage show examples of spatial clusters of sites determined using the clustering algorithm. For the analysis, all spatial clusters from *K* = 1 to *K* = 33 were created

The relationship between spatial scale of grain size and the compositional change over 40 years is shown as a boxplot in Figure 4. Note, when sites are clustered into one group (*K* = 1), there is no variation and so only one distance value for the comparison between years is shown (MH dissimilarity = 0.19). At the finest spatial scale (*K* = 33), mean MH dissimilarity was 0.66. The distribution of dissimilarity values decreases in median value as the clusters get larger (spatial grain) as more sites are pooled together (moving right to left on the *x*‐axis of Figure 4). A decline in median distance begins at approximately *K* = 20, before reaching a local minimum at *K* = 15. For an idea of the spatial scale of pooled sites at this local minimum, see *K* = 15 panel of Figure 5. Following the decrease, the median begins to vary widely as the clusters grow larger in size. In addition, the variance of the distribution, illustrated by the interquartile range, also appears to change with cluster size.

The group *K* = 15 was selected from the visual analysis as a potential candidate for the boundary between domains of scale. The mean MH dissimilarity at *K* = 15 was 0.56. The bootstrapping analysis determined the probability of sampling that mean from the distribution of means sampled form the *K* = 33 data (*n* = 100,000; min = 0.38; 1st quart. = 0.62; median = 0.66; 3rd quart. = 0.70; max. = 0.89) was low (*p* = .062) but not significant at the *α* = 0.05 level. This overall pattern was consistent when MH dissimilarity was substituted for Jaccard's Index, but in that case the bootstrapping analysis of the *K* = 15 distribution was significantly different than the *K* = 33 data (*p* = .028).

## 4 DISCUSSION

The purpose of the spatial analysis presented here was to determine whether and where a transition between assemblage change driven by local factors and change driven by more regional processes occurs. The Muddy Boggy fish assemblage remained similar across 40 years at the global scale. There were 37 species present at both sampling periods, and those present in only one sampling event were rare. Measures of mean alpha diversity corroborate the persistence of diversity over time, and a lack of change in beta diversity and parallel trajectories suggests this system has not experienced increased homogenization of fish taxa since the 1970s. This supports the conclusion of Ross (2013) that most systems tend to have high levels of persistence over time. In addition, these results agree with the synthesis of Matthews and Marsh‐Matthews (2017) which included 40+ years of repeated sampling of drainages in Oklahoma and Arkansas (Matthews & Marsh‐Matthews, 2016; Matthews, Marsh‐Matthews, Cashner, & Gelwick, 2013). The long‐term data presented by those authors suggested high persistence of species assemblages, and when instability occurred from year to year, assemblages tended to return to the original structure.

Local assemblages, however, varied considerably through time. Mean percent dissimilarity was 0.66 at the local scale (*n* = 33 site pairs), and some sites had dissimilarity values as high as 0.99, which indicates complete turnover in species composition. Assemblages changed more on average over the 40‐year period than over a 1‐year period (Figure 2). Although some individual assemblages changed more over 1 year, then others changed over 40 years. While the results suggest stability at the global level, the resolution of the data (i.e., lack of temporal replication) makes interpretations about the local level more difficult. Assemblage change at the individual stream reach is context dependent, and in this case, a range of degrees of change were observed: Some individual reaches contain essentially the same assemblages as 40 years prior, while others have entirely different groups of fishes occupying them. Therefore, the time series presented here cannot be used to support either equilibrium hypothesis, nonequilibrium hypothesis, or loose‐equilibrium hypothesis, but based on the distribution of changes (Figure 2), nonequilibrium seems to be more common.

This result mirrors that of other studies that found variability at the local scale while measuring a lack of change at the global scale (Magurran & Henderson, 2010; Matthews, 1986; Matthews et al., 2013; Ross et al., 1985). Differences in assemblage dynamics over time at broad versus fine spatial scales may be due to the relative importance of different factors which govern the dynamics at these levels (Wiens, 1989). For example, local dynamics might result largely from colonization and extirpation due to processes such as competition and predation, and perturbations such as drought and floods. The dynamics at broader scales may be coupled more with regional species pools and climate.

This study highlights the importance of spatial scale in estimating temporal change in assemblages. There are two apparent declines in median Morisita–Horn dissimilarity going from fine spatial scale to broad spatial scale, the first occurring at *K* = 15 and the second at *K* = 1 (Figure 4). It is not possible to test whether the drop at *K* = 1 was significant due to lack of replication. The drop at *K* = 15 was not statistically significant at *α* = 0.05 for the MH‐Index; however, the sample size (*n* = 15) was small and the difference was marginally significant (*p* = .062). The same discontinuity was observed when using Jaccard's Index on coarser data (presence/absence) and in that case the difference was significant. In addition, discontinuities that represent domains or hierarchies are expected to be followed by unusually high variation (Greig‐Smith, 1979; O'Neill et al., 1986), as illustrated by Figure 4. Therefore, this discontinuity may represent an interesting pattern and scale that should be explored further.

The size of the spatial clusters at *K* = 15 (Figure 5) is roughly the size of the headwater drainages (Stream Order ≤ 3) or tributaries to the main channel of either Clear Boggy or Muddy Boggy Creek. Spatial units of this size may represent a transition between “spatial domains” (Wiens, 1989). For example, individual stream reaches are impacted by biotic, abiotic, and stochastic factors which can lead to instability and lack of persistence. At larger sampling scales (i.e., regions), multiple sites may be linked by dispersal and make up a meta‐community (Leibold et al., 2004; Muneepeerakul et al., 2008) in which changes in assemblage composition at the individual site reach do not necessarily affect persistence of species at the regional scale. However, it has been suggested that instability at fine scales may result in stability at broader scales (DeAngelis & Waterhouse, 1987), owing at least in part to opportunity that instability can create for colonizing species (Wiens, 1977). The discontinuity in assemblage change that occurs at *K* = 15 may represent the transition between the dominance of individual reach factors mentioned above and the dominance of more regional factors at the meta‐community level.

Another study of fish assemblage change over time (Hoeinghaus, Layman, Arrington, & Winemiller, 2003) found discriminant function analysis could assign the identity of a creek (i.e., tributary) based on the fish assemblage found there, which suggested creek‐specific fish assemblages. These creek‐specific assemblages may become isolated by the main channel of the larger drainage where there are obvious changes in habitat, depth, and predator densities which may limit dispersal between creeks. At this spatial scale, dynamics become more stable because the system is partly closed to immigration and emigration, which in part is responsible for change at the scale of an individual stream reach. Most species of fish in southeastern Oklahoma readily move in and out of a 100‐m stream reach over a lifetime, but moving out of a tributary drainage is more difficult (Radinger & Wolter, 2014). Systems such as Brier Creek, OK and Piney Creek, AR, investigated in studies noted above for their persistence and stability (Matthews & Marsh‐Matthews, 2016; Matthews et al., 2013), are much smaller than the Muddy Boggy system but compare closely to the size of the groupings shown at *K* = 15 (i.e., tributary drainages). This evidence suggests assemblages are more predictable when defined as a group of fish occurring within an area much larger than a single stream reach, possibly the size of a 2nd‐ or 3rd‐order tributary drainage.

The results of this study have broad implications which should be considered. First, evidence presented here suggests temporal change in assemblages is scale dependent. Context has long been considered important for understanding community structure (Schlosser, 1991), and many studies have explored how spatial scale affects spatial dynamics (Perkin et al., 2019; Schlosser, 1982), but this study is the first to do so using a continuous spatial gradient of grain size. Second, this test has provided a framework for testing the minimum area required to maximize observed persistence of assemblages through time (Connell & Sousa, 1983), and this information should be considered when defining what the “assemblage” means for future studies of biodiversity in other study systems. For stream fish, the assemblage is often defined as the fish occurring together within a sampled stream reach. However, given the results presented here, perhaps a more accurate representation of an assemblage would include multiple sites from the same tributary drainage “pooled” together (Hitt & Roberts, 2012). And third, this study highlights that small‐scale instability need not result in larger scale changes. It is possible that the instability at smaller spatial scales allows for stability at higher levels by relaxing biotic advantages held by some competitors and predators which creates opportunity for other species to thrive. This point is critical to keep in mind for any biodiversity monitoring program. What scale is being measured and is compositional change at that scale what needs to be prioritized? Finally, compositional changes at the local level could be the result of processes at higher spatial hierarchies (e.g., meta‐community processes at the tributary drainage level). Therefore, future studies of persistence and stability should take scale into consideration to better understand the context that results in various levels of equilibrium (or nonequilibrium).

## CONFLICT OF INTERESTS

None declared.

## AUTHOR CONTRIBUTIONS

ZDZ analyzed the data and wrote the manuscript.

## Data Availability

All data are included as Table S1.
