# Supplementary material for: Temporal dynamics of stream fish assemblages and the role of spatial scale in quantifying change
Source: Ecol Evol. 2020 Jan 2;10(2):952–61. doi: 10.1002/ece3.5954 (PMC6988559; doi:10.1002/ece3.5954)
Supplement: Supplementary file 1 [file ECE3-10-952-s001.docx]

**Table S1.** Species list for 33 sites sampled in 1974 (left) and 2014 (right). Species sorted by abundance in 1974.

| **1974** | | | | | | | | | | | |  | | | | **2014** | | | | | | | | | | | |
| --- | --- | --- | --- | --- | --- | --- | --- | --- | --- | --- | --- | --- | --- | --- | --- | --- | --- | --- | --- | --- | --- | --- | --- | --- | --- | --- | --- |
| **Species common to both sampling times** | | | | | | | | | | | | | | | | | | | | | | | | | | | |
| **No.** | | **Code** | | **Species** | | **Indiv.** | | | **Sites** | |  | | |  | | | **Species** | | **Indiv.** | | | **Sites** | | |  |  |  |
| 1 | NOTBOO | | *Notropis boops* | | | 719 | | 11 | |  | | |  | | | | *Notropis boops* | | | 668 | | | 10 | | |  |  |
| 2 | CYPLUT | | *Cyprinella lutrensis* | | | 654 | | 9 | |  | | |  | | | | *Cyprinella lutrensis* | | | 640 | | | 11 | | |  |  |
| 3 | CYPVEN | | *Cyprinella venusta* | | | 573 | | 13 | |  | | |  | | | | *Cyprinella venusta* | | | 546 | | | 13 | | |  |  |
| 4 | GAMAFF | | *Gambusia affinis* | | | 572 | | 23 | |  | | |  | | | | *Gambusia affinis* | | | 728 | | | 25 | | |  |  |
| 5 | ETHRAD | | *Etheostoma radiosum* | | | 568 | | 19 | |  | | |  | | | | *Etheostoma radiosum* | | | 198 | | | 21 | | |  |  |
| 6 | LYTUMB | | *Lythrurus umbratilis* | | | 291 | | 17 | |  | | |  | | | | *Lythrurus umbratilis* | | | 435 | | | 17 | | |  |  |
| 7 | CAMSPE | | *Campostoma sp.* | | | 279 | | 16 | |  | | |  | | | | *Campostoma sp.* | | | 708 | | | 22 | | |  |  |
| 8 | PIMVIG | | *Pimephales vigilax* | | | 242 | | 9 | |  | | |  | | | | *Pimephales vigilax* | | | 90 | | | 14 | | |  |  |
| 9 | LEPMEG | | *Lepomis megalotis* | | | 193 | | 25 | |  | | |  | | | | *Lepomis megalotis* | | | 361 | | | 24 | | |  |  |
| 10 | LEPCYA | | *Lepomis cyanellus* | | | 177 | | 23 | |  | | |  | | | | *Lepomis cyanellus* | | | 54 | | | 15 | | |  |  |
| 11 | LEPMAC | | *Lepomis macrochirus* | | | 148 | | 21 | |  | | |  | | | | *Lepomis macrochirus* | | | 360 | | | 24 | | |  |  |
| 12 | ICTPUN | | *Ictalurus punctatus* | | | 139 | | 2 | |  | | |  | | | | *Ictalurus punctatus* | | | 11 | | | 5 | | |  |  |
| 13 | PIMNOT | | *Pimephales notatus* | | | 132 | | 11 | |  | | |  | | | | *Pimephales notatus* | | | 144 | | | 8 | | |  |  |
| 14 | NOTSTR | | *Notropis stramineus* | | | 115 | | 5 | |  | | |  | | | | *Notropis stramineus* | | | 36 | | | 2 | | |  |  |
| 15 | FUNSPE | | *Fundulus sp.* | | | 88 | | 8 | |  | | |  | | | | *Fundulus sp.* | | | 8 | | | 2 | | |  |  |
| 16 | NOTNOC | | *Noturus nocturnus* | | | 55 | | 9 | |  | | |  | | | | *Noturus nocturnus* | | | 17 | | | 3 | | |  |  |
| 17 | NOTCRY | | *Notemigonus crysoleucas* | | | 52 | | 13 | |  | | |  | | | | *Notemigonus crysoleucas* | | | 122 | | | 15 | | |  |  |
| 18 | PERSCI | | *Percina sciera* | | | 35 | | 8 | |  | | |  | | | | *Percina sciera* | | | 25 | | | 6 | | |  |  |
| 19 | PIMPRO | | *Pimephales promelas* | | | 32 | | 2 | |  | | |  | | | | *Pimephales promelas* | | | 378 | | | 4 | | |  |  |
| 20 | LABSIC | | *Labidesthes sicculus* | | | 28 | | 3 | |  | | |  | | | | *Labidesthes sicculus* | | | 40 | | | 6 | | |  |  |
| 21 | POMANN | | *Pomoxis annularis* | | | 21 | | 7 | |  | | |  | | | | *Pomoxis annularis* | | | 35 | | | 8 | | |  |  |
| 22 | MICPUN | | *Micropterus punctulatus* | | | 19 | | 10 | |  | | |  | | | | *Micropterus punctulatus* | | | 5 | | | 2 | | |  |  |
| 23 | ETHGRA | | *Etheostoma gracile* | | | 16 | | 2 | |  | | |  | | | | *Etheostoma gracile* | | | 12 | | | 7 | | |  |  |
| 24 | LEPMIC | | *Lepomis microlophus* | | | 13 | | 6 | |  | | |  | | | | *Lepomis microlophus* | | | 49 | | | 10 | | |  |  |
| 25 | MICSAL | | *Micropterus salmoides* | | | 13 | | 9 | |  | | |  | | | | *Micropterus salmoides* | | | 90 | | | 16 | | |  |  |
| 26 | NOTSUT | | *Notropis suttkusi* | | | 12 | | 1 | |  | | |  | | | | *Notropis suttkusi* | | | 640 | | | 4 | | |  |  |
| 27 | ETHSPE | | *Etheostoma spectabile* | | | 8 | | 3 | |  | | |  | | | | *Etheostoma spectabile* | | | 52 | | | 5 | | |  |  |
| 28 | CYPWHI | | *Cyprinella whipplei* | | | 7 | | 2 | |  | | |  | | | | *Cyprinella whipplei* | | | 208 | | | 5 | | |  |  |
| 29 | LEPHUM | | *Lepomis humilis* | | | 6 | | 4 | |  | | |  | | | | *Lepomis humilis* | | | 45 | | | 11 | | |  |  |
| 30 | AMENAT | | *Ameiurus natalis* | | | 5 | | 3 | |  | | |  | | | | *Ameiurus natalis* | | | 4 | | | 2 | | |  |  |
| 31 | PERPHO | | *Percina phoxocephala* | | | 5 | | 3 | |  | | |  | | | | *Percina phoxocephala* | | | 4 | | | 3 | | |  |  |
| 32 | PHEMIR | | *Phenacobius mirabilis* | | | 2 | | 2 | |  | | |  | | | | *Phenacobius mirabilis* | | | 110 | | | 7 | | |  |  |
| 33 | AMEMEL | | *Ameiurus melas* | | | 1 | | 1 | |  | | |  | | | | *Ameiurus melas* | | | 5 | | | 4 | | |  |  |
| 34 | LEPGUL | | *Lepomis gulosus* | | | 1 | | 1 | |  | | |  | | | | *Lepomis gulosus* | | | 3 | | | 3 | | |  |  |
| 35 | MINMEL | | *Minytrema melanops* | | | 1 | | 1 | |  | | |  | | | | *Minytrema melanops* | | | 27 | | | 4 | | |  |  |
| 36 | MOXERY | | *Moxostoma erythrurum* | | | 1 | | 1 | |  | | |  | | | | *Moxostoma erythrurum* | | | 13 | | | 4 | | |  |  |
| 37 | NOTBUC | | *Notropis buchanani* | | | 1 | | 1 | |  | | |  | | | | *Notropis buchanani* | | | 122 | | | 2 | | |  |  |
| **Species not common to both sampling times** | | | | | | | | | | | | | | | | | | | | | | | | | | |  |
| **1974** | | | | | | | | | | |  | | | | **2014** | | | | | | | | | | | |  |
| **No.** | | **Code** | | | **Species** | | **Indiv.** | | **Sites** | |  | | | | **Code** | | | **Species** | | | **Indiv.** | | | **Sites** | | |  |
| 38 | HYBNUC | | Hybognathus nuchalis | | | 16 | | 1 | |  | | | DORCEP | | | | *Dorosoma cepedianum* | | | 2 | | | 1 | | |  |  |
| 39 | ICTFUR | | Ictalurus furcatus | | | 2 | | 1 | |  | | | ETHCHL | | | | *Etheostoma chlorosomum* | | | 1 | | | 1 | | |  |  |
| 40 | NOTVOL | | Notropis volucellus | | | 2 | | 1 | |  | | | HYBAMN | | | | *Hybopsis amnis* | | | 1 | | | 1 | | |  |  |
| 41 | APHSAY | | Aphredoderus sayanus | | | 1 | | 1 | |  | | | LEPOSS | | | | *Lepisosteus osseus* | | | 2 | | | 1 | | |  |  |
| 42 | APLGRU | | Aplodinotus grunniens | | | 1 | | 1 | |  | | | NOTGYR | | | | *Noturus gyrinus* | | | 3 | | | 3 | | |  |  |
| 43 | ESOAME | | Esox americanus | | | 1 | | 1 | |  | | | CHRERY | | | | *Chrosomus erythrogaster* | | | 19 | | | 1 | | |  |  |
| 44 | ETHNIG | | Etheostoma nigrum | | | 1 | | 1 | |  | | | POMNIG | | | | *Pomoxis nigromaculatus* | | | 3 | | | 3 | | |  |  |
| 45 | ICTBUL | | Ictiobus bubalus | | | 1 | | 1 | |  | | |  | | | |  | | |  | | |  | | |  |  |
| 46 | NOTATH | | Notropis atherinoides | | | 1 | | 1 | |  | | |  | | | |  | | |  | | |  | | |  |  |
